# Synonymous substitutions confer the conserved *WPRa4* as a novel target of miR396 in cucumber

**DOI:** 10.1093/hr/uhag036

**Published:** 2026-02-16

**Authors:** Xu Wang, Longlong Zheng, Zhihui Sun, Jiaqi Pan, Ze Li, Chenhao Zhou, Yong He, Zhujun Zhu, Yunmin Xu

**Affiliations:** College of Horticulture, Zhejiang Agriculture and Forestry University, Hangzhou, Zhejiang 311300, China; College of Horticulture, Zhejiang Agriculture and Forestry University, Hangzhou, Zhejiang 311300, China; College of Horticulture, Zhejiang Agriculture and Forestry University, Hangzhou, Zhejiang 311300, China; College of Horticulture, Zhejiang Agriculture and Forestry University, Hangzhou, Zhejiang 311300, China; College of Horticulture, Zhejiang Agriculture and Forestry University, Hangzhou, Zhejiang 311300, China; College of Horticulture, Zhejiang Agriculture and Forestry University, Hangzhou, Zhejiang 311300, China

## Abstract

As an evolutionarily conserved microRNA (miRNA), miR396 regulates plant growth by integrating developmental and environmental signals. In the present study, *CsaWPRa4*, a *WEB1* (*Weak Chloroplast Movement under Blue Light 1*)/*PMI2* (*Plastid Movement Impaired 2*)-*related protein* (*WPR*) family member, was predicted to be a novel target gene of CsamiR396 in cucumbers. WPRa4 is a highly conserved protein in plants. Interestingly, bioinformatic analysis showed that *WPRa4* acts as a conserved target gene of miR396 in cucumber and its related species in cucurbits, but not in other plants. The miR396 binding site is located within the coding region of the AAK(K/R)AVE motif in WPRa4, and it evolved by synonymous substitutions in cucurbits. Negative regulation of *CsaWPRa4* by CsamiR396 was confirmed by reverse transcription‑quantitative polymerase chain reaction (RT‑qPCR), luciferase assay, gene overexpression, and tobacco ringspot virus (TRSV)-based gene silencing analysis. The subcellular localization assay showed that CsaWPRa4 was localized to both the cell periphery and nuclear periphery. Thereafter, *Csawpra4* mutants were generated using CRISPR/Cas9-mediated gene editing. Chloroplast- and flower morphogenesis-related genes were altered, resulting in altered photosynthetic traits and flower morphogenesis in *Csawpra4* mutants. In summary, our results showed that *WPRa4* evolved as a novel target of miR396 through synonymous substitutions in cucurbits, uncovering the role of synonymous substitutions in genome evolution and providing a new perspective on miRNA–target evolutionary processes in plants.

## Introduction

MicroRNAs (miRNAs) are small noncoding RNAs that negatively regulate target genes via transcript cleavage or translational inhibition. As an ancient miRNA, miR396 negatively regulates *GROWTH*-*REGULATING FACTOR* (*GRF*) family genes, and the miR396–GRF pathway is conserved in plants [1, 2]. *In vivo*, the GRF transcription factor interacts with the GRF interacting factor (GIF), a transcriptional coactivator, to form the GRF–GIF complex, which promotes plant growth by enhancing cell proliferation [3]. Accordingly, the overexpression of miR396 results in smaller organs, whereas the overexpression of *GRFs* results in larger organs in plants [1, 4, 5]. Since the miR396–GRF pathway conservatively regulates cell proliferation, it has been manipulated to enhance regeneration efficiency during transformation in plants [6–8].

In addition to the conserved *GRF* target genes, miR396 also regulates species-specific target genes in plants. For instance, in Arabidopsis, miR396 targets *bHLH74*, which encodes a basic helix–loop–helix (bHLH) transcription factor, to regulate vein pattern [9], root length [10], and flowering [11]. In *Catharanthus roseus*, the miR396-targeted *SHORT VEGETATIVE PHASE* (*SVP*) is required to repress flowering and is related to the development of abnormal flower symptoms by the Phyllody Symptoms1 Effector [12]. Additionally, miR396 targets *1-aminocyclopropane-1-carboxylic acid oxidase* (*ACO*) to regulate cold tolerance in *Poncirus trifoliata* [13], whereas miR396 targets *tetratricopeptide repeat-like superfamily protein* (*TPR*) to regulate cold tolerance in *Cucumis sativus* [14].

miR396 is encoded by different loci in plants, with two loci (*AtMIR396A*-*B*) in *Arabidopsis thaliana* [4], eight loci (*OsMIR396A*-*F*) in *Oryza sativa* [15], and five loci (*CsaMIR396A*-*E*) in *C. sativus* [16]. In plants, miR396 is conservatively regulated by an intrinsic age-related signal. For instance, miR396 accumulates with increasing leaf age, resulting in a decrease in *GRFs*, and the miR396–GRF pathway regulates age-dependent cell proliferation [1, 17]. Additionally, miR396 expression is regulated by environmental signals, such as drought stress, high salinity, and UV-B [2, 18]. In summary, miR396 regulates plant growth by integrating developmental and environmental signals.

WEB1 (Weak Chloroplast Movement under Blue Light 1)/PMI2 (Plastid Movement Impaired 2)-related protein (WPR) is a plant-specific coiled-coil protein family, which contains a WEMBL domain [19]. The WPR proteins were phylogenetically subdivided into four groups. For instance, the WPR family includes 14 members in Arabidopsis: WEB1 (WEB1, WEL1, WEL2, and WEL3), PMI2 (PMI2 and PMI15), WPRa (WPRa1, WPRa2, WPRa3, and WPRa4), and WPRb (WPRb1, WPRb2, WPRb3, and WPRb4) [19]. WEB1, PMI2, and PMI15 affect chloroplast actin filaments (cp-actin filaments) to regulate the chloroplast photorelocation movement response in Arabidopsis [19–21]. *WPRa4*, also known as *TOUCH-REGULATED PHOSPHOPROTEIN1* (*TREPH1*), localizes near plastids and interacts with the plastidic translocon component [22]. The WPRa4 protein is rapidly phosphorylated under touch stimulation in Arabidopsis, and it plays a role in touch-induced bolting delay and touch-induced gene expression, such as *CALMODULIN-LIKE38* (*CML38*), *ETHYLENE RESPONSE FACTOR11* (*ERF11*), and *JASMONATE-ZIM-DOMAIN PROTEIN7* (*JAZ7*) [23]. However, another study showed no significant alterations in touch-induced gene expression between WT and *wpra4* mutants in Arabidopsis [24].

Synonymous substitution (synonymous mutation) is a change in the coding DNA sequence that does not alter the encoded amino acids. Most synonymous substitutions are thought to be biologically silent, with a few exceptions [25]. Once known as ‘silent’ mutations, synonymous substitutions are increasingly attracting interest of biologists [25–27]. Our results showed that *WPRa4*, a conserved *WPR* family member in plants, evolved as a novel target of miR396 through synonymous substitutions in cucumber and its related species in cucurbits. Additionally, our results confirmed that synonymous substitutions could contribute to the evolution of miRNA–target interactions and affect gene function via post-transcriptional regulation in plants.

## Results

### *CsaWPRa4* is predicted to be a novel target of CsamiR396 in cucumber

**Figure 1:**
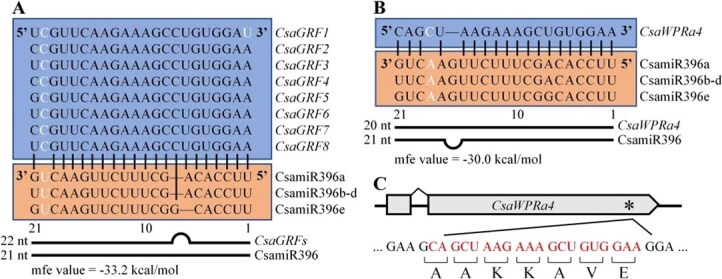
*CsaWPRa4* is predicted as a novel target gene of miR396 in cucumber. (A) Alignment of miR396 and its binding site in *CsaGRFs*. (B) Alignment of miR396 and its binding site in *CsaWPRa4*. Mismatch nucleotides are marked in white. The mfe value refers to the minimum free energy hybridization of miR396 and its target gene. (C) Schematic illustration of the putative miR396 binding site within *CsaWPRa4* coding region. The putative miR396 binding site is marked with an asterisk, and its nucleotide sequences are marked in red.

*GRFs* are conserved target genes of miR396. miR396 also harbors species-specific target genes in plants. The CsamiR396–CsaGRF pathway in cucumbers was identified in our previous study [16]. Similar to *CsaGRFs*, CsaV3_3G004170 was identified as a CsamiR396 well-matched gene by Blastn analysis (Fig. 1). CsaV3_3G004170 encodes a WPR family protein and was named CsaWPRa4 according to its homology (AtWPRa4) in Arabidopsis (Fig. S1).

Thereafter, the CsamiR396–CsaWPRa4 interaction was compared with that of the conserved CsamiR396–CsaGRFs. CsamiR396 contained 21 nucleotides and matched a 20-nt sequence of *CsaWPRa4*, whereas it matched a 22-nt sequence of *CsaGRFs*, resulting in a bulge within CsamiR396 in the CsamiR396–CsaWPRa4 interaction, and a bulge within *CsaGRFs* in the CsamiR396–CsaGRF interaction (Fig. 1A and B). The CsamiR396–CsaWPRa4 interaction harbors a mismatch at the 18th nucleotide position, while the CsamiR396–CsaGRF interaction harbors a mismatch at the 20th nucleotide position. The minimum free energy (mfe) hybridization value of the CsamiR396–CsaWPRa4 and CsamiR396–CsaGRF interactions were −30.0 and −33.2 kcal/mol, respectively (Fig. 1A and B). Moreover, the CsamiR396 binding site was located within the coding region of *CsaWPRa4* and encoded an AAKKAVE motif in cucumber (Fig. 1C). In summary, the WPR family protein coding gene *CsaWPRa4* was predicted to be a novel target of CsamiR396 in cucumbers.

### *WPRa4* evolves as a conserved target gene of miR396 by synonymous substitutions in cucurbits

CsaWPRa4 is a member of the WPR family belonging to the WPRa group. Here, the homologs of CsaWPRa4 in three dicotyledons (*A. thaliana*, *Solanum lycopersicum*, *Glycine max*) and one monocotyledon (*O. sativa*) were identified using Blastp analysis, and their sequences were aligned using ClustalX2. As shown in Fig. S2, WPRa4 is a highly conserved WPR family member in plants, and the AAK(K/R)AVE motif is conservatively located within the C-terminus of the WEMBL domain.

**Figure 2:**
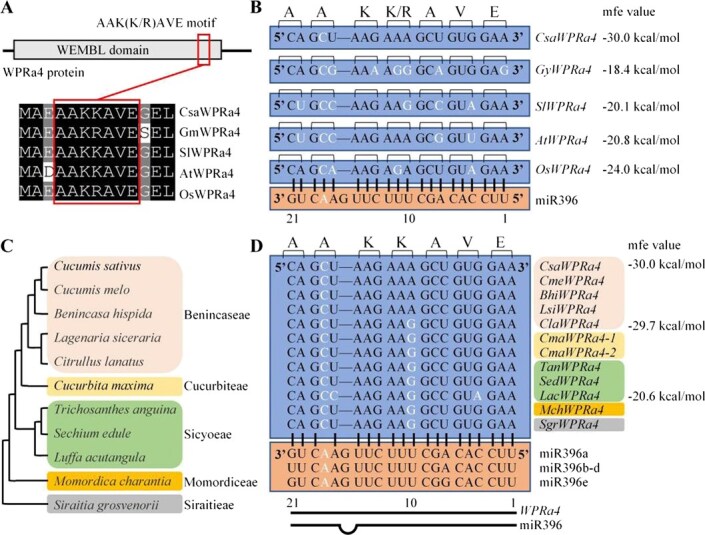
*WPRa4* is a conserved target gene of miR396 in cucurbits. (A) The conserved AAK(K/R)AVE motif of CsaWPR4a and its homologs in plants. (B) Alignment of miR396 and its putative binding site in *WPRa4* in plants. *Csa*, *Gm*, *Sl*, *At*, and *Os* represent *C. sativus*, *G. max*, *S. lycopersicum*, *A. thaliana*, and *O. sativa*, respectively. (C) Phylogenetic relationship of cucurbit plants (modified from Guo *et al.* [28]). (D) Alignment of miR396 and its putative binding site in *WPRa4* in cucurbits. *Csa*, *Cme*, *Bhi*, *Lsi*, *Cla*, *Cma*, *Tan*, *Sed*, *Lac*, *Mch*, and *Sgr* represent *C. sativus*, *Cucumis melo*, *Benincasa hispida*, *Lagenaria siceraria*, *Citrullus lanatus*, *Cucurbita maxima*, *Trichosanthes anguina*, *Sechium edule*, *Luffa acutangula*, *Momordica charantia*, and *Siraitia grosvenorii*, respectively. The mfe value refers to the minimum free energy hybridization of miR396 and its target gene. Mismatch nucleotides are marked in white.

In cucumber, the coding sequence of the AAK(K/R)AVE motif in CsaWPRa4 acts as a CsamiR96 binding site (Fig. 1C). AAK(K/R)AVE is a conserved motif in plants (Fig. S2, Fig. 2); the miR396–WPRa4 interaction was investigated in different plants. Compared with the CsamiR396–CsaWPRa4 interaction, six, five, four, and three additional mismatch nucleotides were presented in the GymiR396–GyWPRa4 interaction, SlmiR396–SlWPRa4 interaction, AtmiR396–AtWPRa4 interaction, and OsmiR396–OsWPRa4 interaction, resulting in an increase of the mfe value to −18.4, −20.1, −20.8, and −24.0 kcal/mol, respectively (Fig. 2B). In summary, compared to *CsaWPRa4*, changes in nucleotides within the coding sequence of the AAK(K/R)AVE motif destroyed the match between miR396 and *WPRa4* in other plants.

Interestingly, these nucleotides are located within the second or third position of the genetic code (Fig. 2B). Compared with *CsaWPRa4*, the changed nucleotides (1st, 4th, 7th, 10th, 13th, 17th, and 20th) in *WPRa4* in other plants, which were located within the third position of the genetic code, did not alter the amino acid sequences due to the degeneracy of the genetic code. A changed nucleotide (11th) in *WPRa4* of *G. max* and *O. sativa*, located within the second position of the genetic code, altered Lys to Arg. Lys and Arg are functionally similar due to their shared positive charges. In summary, the conserved plant *WPRa4* acted as a target gene of miR396 in cucumbers, but not in other plants, and synonymous substitutions contributed to this change.

To gain insight into whether the miR396–WPRa4 interaction was specifically present in cucumber, alignment between miR396 and *WPRa4* was performed in cucumber and its related species in cucurbits. The phylogenetic relationships of cucurbit plants were modified from a previous study [28] (Fig. 2C). Sequence alignment showed that *WPRa4* acted as a conserved miR396 target gene in cucurbits (Fig. 2D). Compared with the later diverging lineage (Benincaseae), an additional mismatch nucleotide (10th) was presented in the miR396–WPRa4 interaction in the early diverging lineages (Cucurbiteae, Sicyoeae, Momordiceae, and Siraitieae) (Fig. 2D). However, the miR396–LacWPRa4 interaction harbored four mismatch nucleotides, and its mfe value was increased to −20.6 kcal/mol. In summary, these results suggest that the plant’s conserved *WPRa4* evolved as a conserved target gene of miR396 through synonymous substitutions in cucurbits.

### Negative regulation of *CsaWPRa4* by CsamiR396 in cucumber

**Figure 3:**
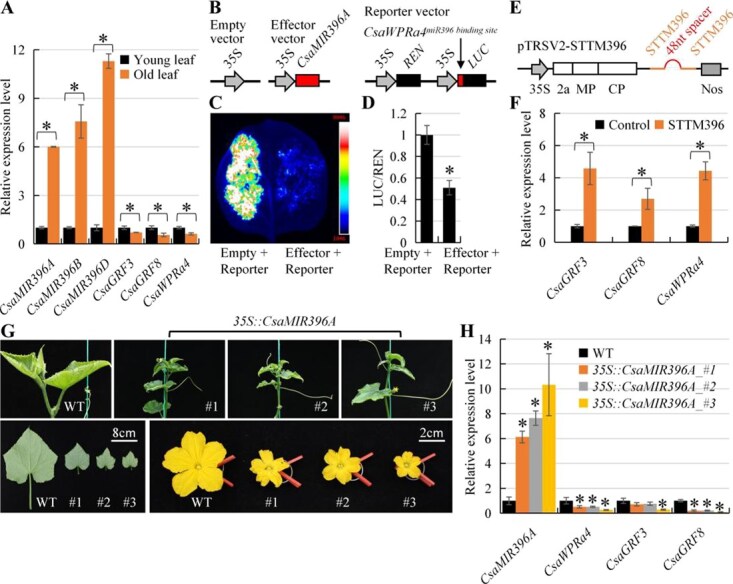
miR396 negatively regulates *CsaWPRa4 in vitro* and *in vivo*. (A) RT-qPCR detection of *CsaMIR396*, *CsaGRFs*, and *CsaWPRa4* in cucumber leaves. (B) Schematic illustration of effector vectors and reporter vector in luciferase assay. (C) Luciferase assay of *35S::CsaWPRa4^miR396 binding site^-LUC* under coexpression of control or *35S::CsaMIR396A* vector. (D) Relative *LUC*/*REN* ratio. *LUC*/*REN* ratio of control (Empty vector+Reporter vector) is normalized as 1. (E) Schematic illustration of TRSV-mediated silencing of miR396 in cucumber. (F) RT-qPCR detection of *CsaGRFs* and *CsaWPRa4* in control and miR396-silenced cucumber. (G) Phenotypes of *35S::CsaMIR396A* cucumber. (H) RT-qPCR detection of *CsaMIR396A*, *CsaWPRa4*, *CsaGRF3*, and *CsaGRF8* in WT and *35S::CsaMIR396A* cucumber. Asterisks denote statistical significance from control (or WT) at *P* < 0.01 using Student’s *t*-test. Data are mean ± SD from three technical replicates in a representative experiment with three biological replicates.

In this study, the negative regulation of *CsaWPRa4* by CsamiR396 was investigated both *in vitro* and *in vivo*. miR396 is conservatively regulated by an intrinsic age signal in plants [1, 17]; according to it, CsamiR396, *CsaGRFs*, and *CsaWPRa4* in young and old leaves of cucumbers were detected using reverse transcription‑quantitative polymerase chain reaction (RT‑qPCR). Compared with young leaves, the expression levels of *CsaMIR396A*, *CsaMIR396B*, and *CsaMIR396D* were increased, whereas *CsaWPRa4*, *CsaGRF3*, and *CsaGRF8* were decreased in old leaves, as expected (Fig. 3A).

Next, the negative regulation of *CsaWPRa4* by CsamiR396 was investigated using the pGreen II 62-SK/pGreenII 0800-LUC system. The effector vectors included the control vector and *35S::CsaMIR396A* vector, whereas the reporter vector was *35S::CsaWPRa4^miR396 binding site^-LUC*. The transient expression assay showed that the luciferase activity of *35S::CsaWPRa4^miR396 binding site^-LUC* was clearly decreased by the *35S::CsaMIR396A* effector compared to that of the control effector (Fig. 3B–D).

The negative regulation of *CsaWPRa4* by CsamiR396 was investigated using tobacco ringspot virus (TRSV)-based silencing of CsamiR396 in cucumbers. Compared with the control cucumber, *CsaWPRa4*, *CsaGRF3*, and *CsaGRF8* were upregulated 4.4-, 4.6-, and 2.7-fold, respectively, in CsamiR396-silenced cucumbers (Fig. 3E and F), suggesting that the regulation efficiency was similar between the CsamiR396–CsaGRF interaction and CsamiR396–CsaWPRa4 interaction in cucumbers. Moreover, *CsaMIR396A-*overexpression cucumbers (*35S::CsaMIR396A*) were also generated (Fig. 3G). RT-qPCR results showed that *CsaMIR396A* was overexpressed, as expected, whereas *CsaWPRa4*, *CsaGRF3*, and *CsaGRF8* were downregulated in *35S::CsaMIR396A* cucumbers compared to wild-type (WT) cucumbers (Fig. 3H).

In summary, the negative correlation between CsamiR396 and *CsaWPRa4*, luciferase assay *in vitro*, TRSV-based silencing of CsamiR396 *in vivo*, and *CsaMIR396A* overexpression *in vivo* confirmed the negative regulation of *CsaWPRa4* by CsamiR396 in cucumbers.

### Subcellular localization of CsaWPRa4

**Figure 4:**
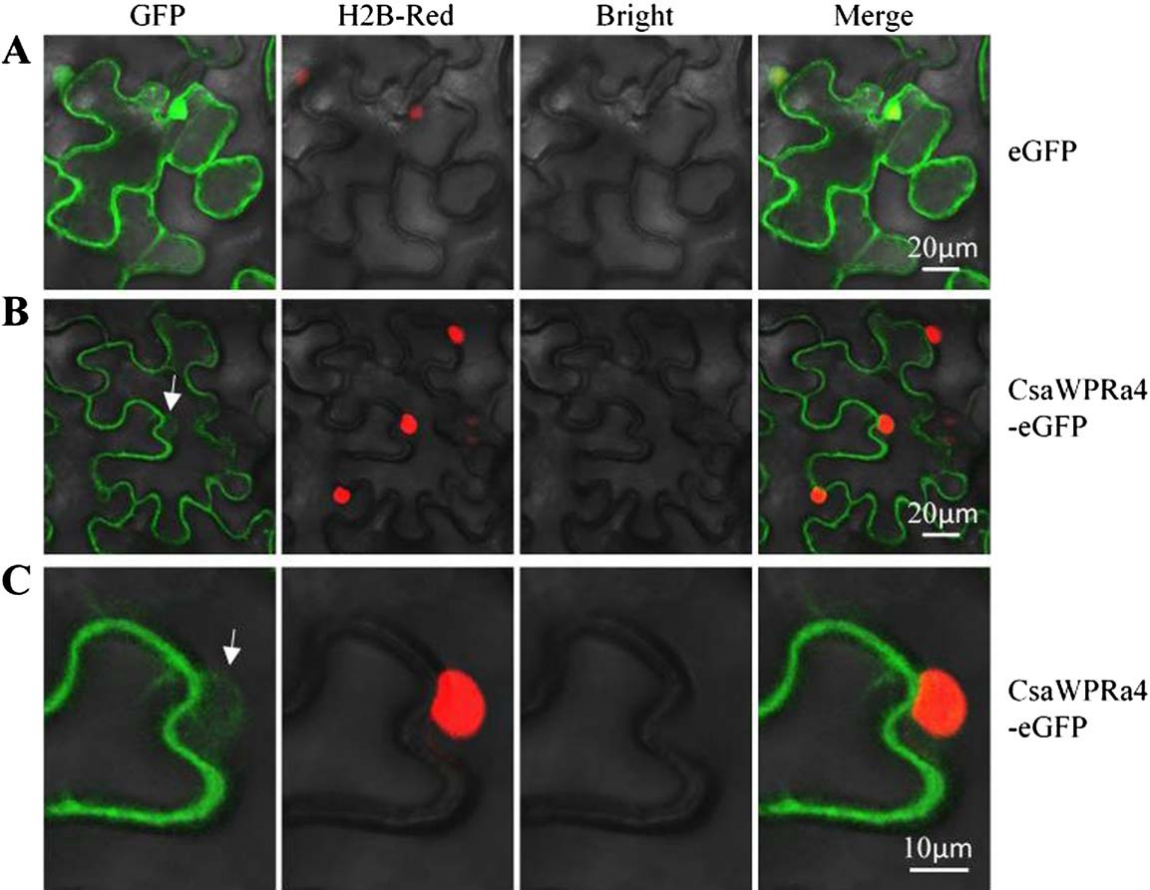
Subcellular localization of CsaWPRa4. (A) Subcellular localization of eGFP. (B) Subcellular localization of the CsaWPRa4-eGFP fusion protein. (C) Enlarged view of CsaWPRa4-eGFP subcellular localization from (B). Nuclear periphery localization of CsaWPRa4-eGFP are marked with white arrows in (B) and (C). H2B-Red as a marker of the cell nucleus.

ePlant predictions (https://bar.utoronto.ca/eplant/) showed that AtWPRa family members exhibit diverse subcellular localizations in Arabidopsis (Fig. S3). AtWPRa1 and AtWPRa2 were localized to the cytosol, AtWPRa3 was localized to both the cytosol and chloroplast, and AtWPRa4 was localized to the plasma membrane (Fig. S3). Recently, subcellular localization showed that AtWPRa4-YFP was localized to both the chloroplast and the plasma membrane in Arabidopsis [22]. In this study, the subcellular localization of CsaWPRa4 was investigated, and the results showed that CsaWPRa4-eGFP was localized in both the cell periphery and nuclear periphery (Fig. 4).

### *CsaWPRa4* regulates chloroplast activity and flower morphogenesis in cucumber

**Figure 5:**
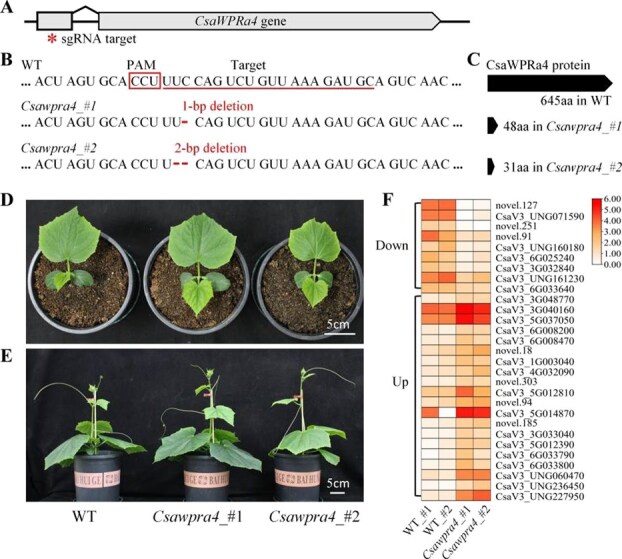
CRISPR/Cas9-generated *Csawpra4* mutant in cucumber. (A) The sgRNA target site for CRISPR/Cas9-generating mutation in *CsaWPRa4*. (B) and (C) The information of two Del- mutations of *CsaWPRa4* (*Csawpra4_#1*, *Csawpra4_#2*). (D) and (E) 15- and 28-day-old WT, *Csawpra4_#1*, and *Csawpra4_#2* plants. (F) The DEGs in *Csawpra4* mutant.

To gain insights into the role of *CsaWPRa4* in cucumbers, CRISPR/Cas9-generated mutations were produced. Here, two independent null mutations (*Csawpra4_#1* and *Csawpra4_#2*), which caused a frame shift and premature translation termination, were generated (Fig. 5A–C). Phenotypic analysis showed that *Csawpra4_#1* and *Csawpra4_#2* exhibited normal developmental processes comparable to those of the WT (Fig. 5D and E). The transcriptomes of WT, *Csawpra4_#1*, and *Csawpra4_#2* seedlings were then compared. Twenty-nine transcripts were altered, 20 of which were upregulated, while nine were downregulated in *Csawpra4* mutants compared to the WT (Fig. 5F, Table S1). Gene annotations showed that 20.7% of the altered transcripts were related to chloroplasts, and three altered transcripts were related to flower morphogenesis (Table S1).

**Figure 6:**
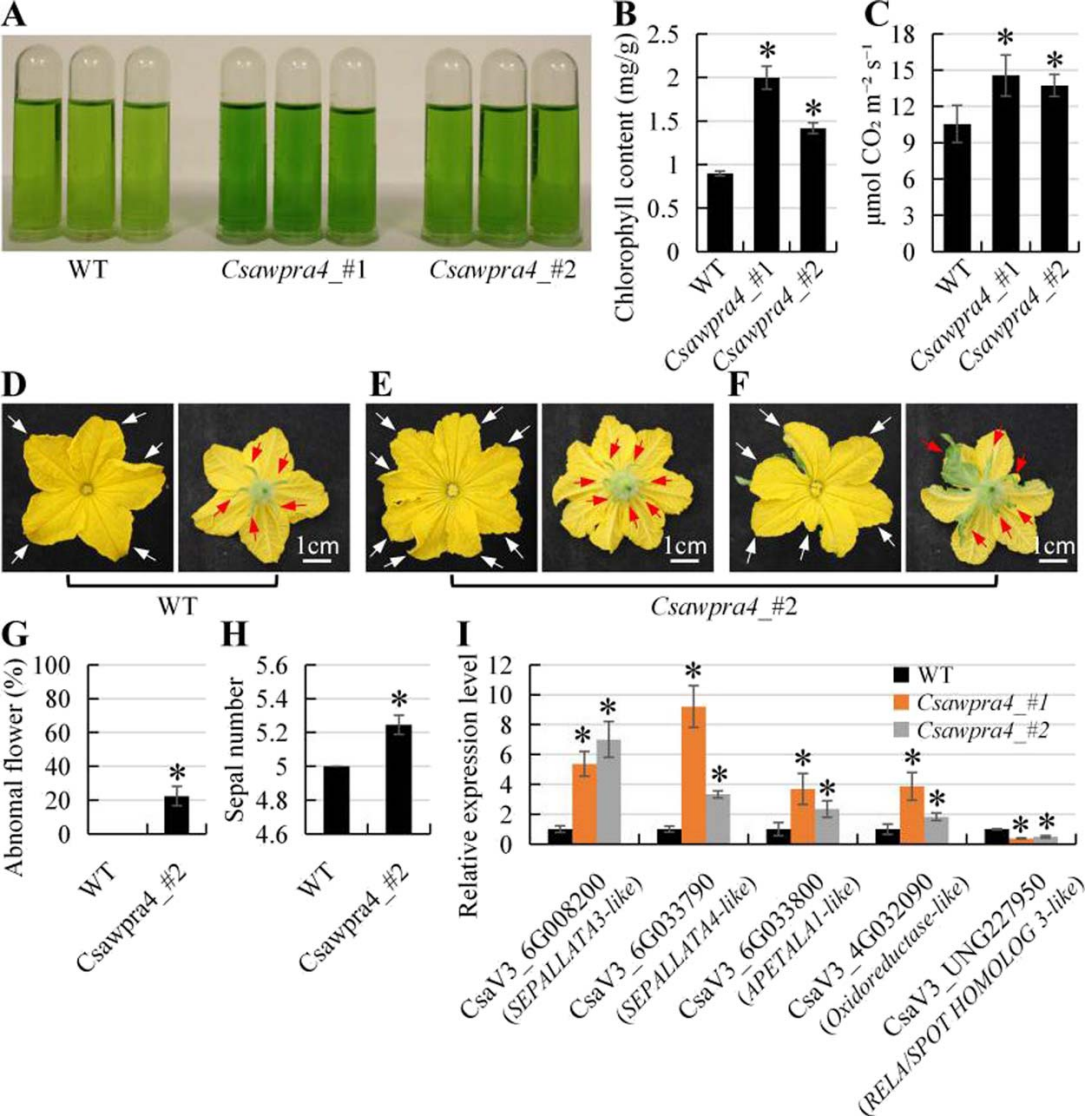
The defective phenotypes of *Csawpra4* mutant in cucumber. (A) and (B) Detection of chlorophyll content. (C) Photosynthetic rate. (D–F) Flower phenotype. Cucumber seedlings are planted in a greenhouse under natural light. Petal and sepal are marked with white arrows and red arrows, respectively. (G) The ratio of abnormal flowers. Data are mean ± SD from *n* = 400 flowers. (H) Sepal number. Data are mean ± SD from *n* = 300 flowers. (I) RT-qPCR confirmation results of DEGs in *Csawpra4* mutant. Asterisks denote statistical significance from WT at *P* < 0.01 using Student’s *t*-test.

Changes in two chloroplast-related genes (CsaV3_4G032090 and CsaV3_UNG227950) and three flower morphogenesis-related genes (CsaV3_6G008200, CsaV3_6G033790, and CsaV3_6G033800) were confirmed using RT-qPCR (Fig. 6I). CsaV3_4G032090 (encoding a chloroplast-localized oxidoreductase-like protein) and CsaV3_UNG227950 (encoding a chloroplast-localized RELA/SPOT HOMOLOG 3-like protein) were upregulated and downregulated in *Csawpra4* mutants, respectively. Phenotypic analysis showed that the loss-of-function of *CsaWPRa4* did not affect the number and size of chloroplasts in the mesophylls (Fig. S4). However, the chlorophyll content and photosynthetic rate were altered in *Csawpra4* mutants (Fig. 5A, B, Fig. S5). Three flower morphogenesis-related genes (CsaV3_6G008200, *SEPALLATA 3-like*; CsaV3_6G033790, *SEPALLATA 4-like*; CsaV3_6G033800, *APETALA1-like*) were upregulated in *Csawpra4* mutants compared to WT cucumbers (Figs 5F and 6I), and *Csawpra4* mutants produced abnormal flowers (increased petal number and sepal number, leaf-like petals) under natural light conditions (Fig. 5D–H). In summary, these results suggested that *CsaWPRa4* plays a role in regulating chloroplast activity and flower morphogenesis in cucumber.

### *De novo* gain-of-function of the AtmiR396–AtWPRa4 pathway by synonymous substitutions in Arabidopsis

**Figure 7:**
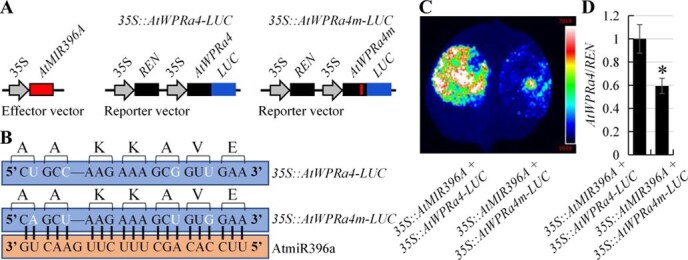
*De novo* gain-of-function of the AtmiR396a–AtWPRa4 pathway by synonymous substitutions. (A) Schematic illustration of effector vector (*35S::AtMIR396A*) and reporter vectors (*35S::AtWPRa4-LUC* and *35S::AtWPRa4m-LUC*) in luciferase assay. *AtWPRa4-LUC* is the fusion expression of *AtWPRa4* and *LUC*. *AtWPRa4m* represents the synonymous substitutions of miR396 binding site within *AtWPRa4*. (B) Synonymous substitutions of the miR396 binding site within *AtWPRa4*. Synonymous substituted nucleotides are marked in white. (C) Luciferase assay of reporter vectors (*35S::AtWPRa4-LUC* and *35S::AtWPRa4m-LUC*) under coexpression of *35S::AtMIR396A* vector. (D) Relative *AtWPRa4*/*REN* ratio. *AtWPRa4*/*REN* ratio of control (*35S::AtMIR396A* + *35S::AtWPRa4-LUC*) is normalized as 1. Asterisks denote statistical significance from control at *P* < 0.01 using Student’s *t*-test. Data are mean ± SD from three technical replicates in a representative experiment with four biological replicates.

*WPRa4* is a highly conserved WPR family member in plants and has evolved as a novel target gene of miR396 through synonymous substitutions in cucurbits (Figs 2 and 3). Here, *de novo* gain-of-function of the AtmiR396–AtWPRa4 pathway by synonymous substitutions was performed in Arabidopsis. *AtWPRa4* harbored the GCU GCC AAG AAA GCG GUU GAA sequence (encoding the AAKKAVE motif), which was substituted with a GCA GCU AAG AAA GCU GUG GAA sequence (mimicking the sequence in cucurbits, encoding the AAKKAVE motif) in *AtWPRa4m* (Fig. 7B). Thereafter, the effector vector (*35S::AtMIR396A*) and reporter vectors (*35S::AtWPRa4-LUC*, *35S::AtWPRa4m-LUC*) were constructed (Fig. 7A), and then AtmiR396–AtWPRa4 and AtmiR396–AtWPRa4m interactions were compared using a luciferase assay and RT-qPCR detection. As shown in Fig. 7C and D, compared to *AtWPRa4*, synonymous substitutions resulted in *AtWPRa4m* acting as a target of AtmiR396. These results showed that the introduction of cucurbit-specific synonymous substitutions into *AtWPRa4* resulted in its targeting by AtmiR396 in Arabidopsis.

## Discussion

### The role of the CsamiR396–CsaGRF/CsaWPRa4 pathway in cucumber

As an ancient miRNA, miR396 is encoded by multiple *MIR396* loci in the genome. Thus, miR396 can integrate different signals by its multiple *MIR396* loci in plants [2]. *GRFs* act as conserved target genes of miR396 and regulate cell proliferation to determine organ size in plants [1, 17]. The miR396–GRF pathway primarily functions in plant size determination under developmental and environmental signals.

**Figure 8:**
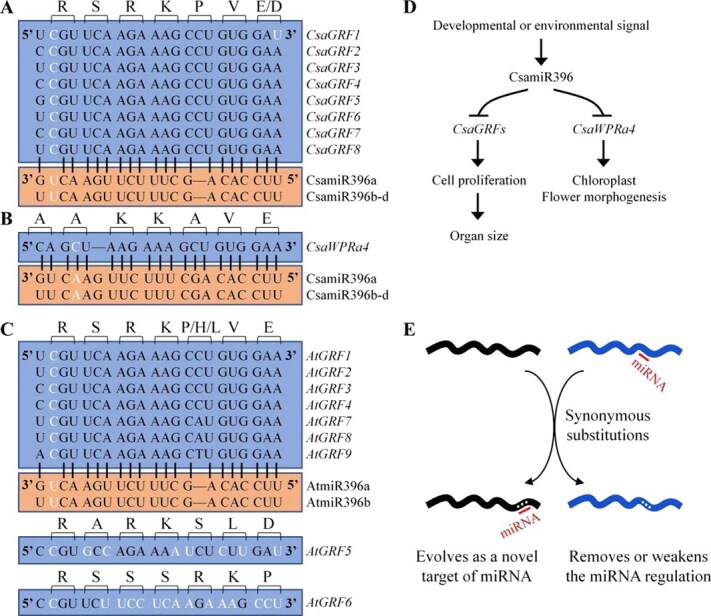
The change of miR396’s target genes in plants. (A) Alignment of miR396 and its binding site in *CsaGRFs* in cucumber. (B) Alignment of miR396 and its binding site in *CsaWPRa4* in cucumber. (C) Alignment of miR396 and its binding site in *AtGRFs* in Arabidopsis. Mismatch nucleotides are marked in white. (D) The role of CsamiR396–CsaGRF/CsaWPRa4 in cucumber. CsamiR396 integrates the intrinsic developmental signal and the extrinsic environmental signal to regulate organ size through *CsaGRF*, while it may regulate chloroplast activity and flower morphogenesis through *CsaWPRa4* in cucumber. (E) Schematic illustration of the role of synonymous substitution in altering miRNA–target gene interactions to contribute genome evolution in plants.

In plants, the WPR family is comprised of four groups: WEB1, PMI2, WPRa, and WPRb [19]. WPR family members were first identified as chloroplast photorelocation movement regulators in Arabidopsis, such as WEB1 from the WEB1 group [21] and PMI2 and PMI15 from the PMI2 group [20]. However, single or double mutants of WPR family members from the WPRa and WPRb groups exhibit normal chloroplast movements, suggesting no involvement or redundant functions of the WPRa and WPRb group genes in the chloroplast photorelocation response [19]. Interestingly, WPRa4 (also named TREPH1) acts as a touch-induced phosphorylation protein, and touch-response genes and touch-induced bolting delays are defective in *wpra4* mutant in Arabidopsis [22, 23]. However, another study showed no significant alterations in touch-induced gene expression between WT and *wpra4* mutants in Arabidopsis [24]. In this study, the conserved *CsaWPRa4* was confirmed to be a novel target of CsamiR396 in cucumbers (Fig. 3). Our results showed that CsaWPRa4 was localized to both the cell periphery and nuclear periphery (Fig. 4). RNA sequencing (RNA-seq) analysis showed that differentially expressed genes (DEGs) in the *Csawpra4* mutant were enriched in chloroplast-related genes, and chlorophyll content and photosynthetic traits were altered in the *Csawpra4* mutant (Table S1, Fig. 6). Additionally, there were changes in flower morphogenesis-related genes in the *Csawpra4* mutant, resulting in flower morphogenesis defects (Table S1, Fig. 6). The role of the CsamiR396–CsaGRF/CsaWPRa4 pathway in cucumbers is discussed and summarized in Fig. 8D. CsamiR396 integrates developmental and environmental signals to regulate organ size through its conserved *CsaGRF* targets and regulates chloroplast activity and flower morphogenesis through its specific *WPRa4* target, which may have contributed to the evolution of cucurbit plants.

### Synonymous substitutions alter miRNA–target gene interactions to influence cellular processes in plants

Most synonymous substitutions (synonymous mutations) in the genome are thought to be biologically silent, with a few exceptions [25]. Recently, synonymous substitutions, once known as ‘silent’ mutations, have attracted the interest of biologists [25–27]. Our results showed that synonymous substitutions within a conserved AAK(K/R)AVE motif led to *WPRa4* becoming a novel target of miR396 in cucurbits, resulting in the post-transcriptional regulation of *CsaWPRa4* by CsamiR396 (Figs 2 and 3).

Here, we investigated the role of synonymous and nonsynonymous substitutions in altering miRNA-target interactions in plants. In cucumber, all eight *GRFs* acted as target genes of miR396 (Fig. 8A), whereas nine *GRFs* were present in Arabidopsis, and two of them (*AtGRF5* and *AtGRF6*) were not the target genes of miR396 (Fig. 8C). Sequence alignment suggested that nonsynonymous substitutions within the miR396 binding site destroy the miR396–GRF interaction in *AtGRF5* and *AtGRF6* (Fig. 8C). In grapevines, the compact inflorescence architecture is regulated by the VvmiR396–VvGRF4 pathway. Interestingly, within the miR396 binding site, a synonymous substitution (18th position) in the 1–86 cultivar and a nonsynonymous substitution (16th position) in the M171 cultivar (Fig. S6) individually destroy the VvmiR396–VvGRF4 interaction, and produced a loose inflorescence architecture [29].

miR156–SPL is another conserved gene pathway in plants. miR156 is highly expressed during the juvenile phase, and its abundance declines gradually during plant development, releasing its *SPL* target genes [30, 31]. A cucurbit-conserved nonsynonymous substitution (11th position) within the miR156 binding site destroys the miR156–SPL7 interaction (Fig. S7A), resulting in high expression of *CsaSPL7* during the juvenile phase in cucumbers; however, its biological significance remains unknown [32]. A nonsynonymous substitution (15th position) within the miR156 binding site interferes the OsmiR156–OsSPL14 interaction (Fig. S7B), which contributes to the ideal plant architecture domestication in rice [33].

In summary, together with nonsynonymous substitutions, synonymous substitutions can influence cellular processes by altering miRNA–target gene interaction (Fig. 8E). On the one hand, synonymous substitution can give rise to novel miRNA–target gene interactions, leading to regulation of the novel target gene by miRNA at the post-transcriptional level. In contrast, synonymous substitution may disrupt preexisting miRNA–target gene interactions, thereby releasing the gene from miRNA-mediated regulation.

## Materials and methods

### Plant materials and growth condition

Cucumber inbred lines (NT1, NT16, and Jinyan) were used in this study. The NT1 inbred line was used for gene detection, the NT16 inbred line for TRSV-based gene silencing assay, and the Jinyan inbred line for CRISPR/Cas9-based gene editing and gene overexpression assays. NT16 is a TRSV-susceptible inbred line [34], while Jinyan is a transgenic inbred line used by Biorun BioSciences (Wuhan, China). NT16 cucumber seedlings for TRSV-based gene silencing assays were planted in a growth chamber with long-day (16 h day at 22°C, 8 h night at 22°C, 18 000 Lx) conditions, whereas NT1 and Jinyan cucumber seedlings were planted in a greenhouse under natural light in autumn 2024 or autumn 2025 (Hangzhou, China).

### Gene annotation and phylogenetic analysis

To annotate of WPRa4 in plants, BLASTP analysis was performed using AtWPRa4 (AT5G55860) as an inquiry sequence, and its homologs in cucurbits, soybean (*G. max*), tomato (*S. lycopersicum*), and rice (*O. sativa*) were identified. Multiple sequence alignments were performed using ClustalX2 and then processed with GeneDoc software. MEGA 7.0 software was employed to construct a phylogenetic tree using the maximum likelihood method.

### miRNA hybridization site analysis

The hybridization site of miR396 within a putative target gene was analyzed using RNAhybrid software (https://bibiserv.cebitec.uni-bielefeld.de), and the mfe value was calculated.

### RNA extraction and RT-qPCR detection

Cucumber seedlings were collected and stored at −80°C, and total RNA was extracted using TRIzol reagent (Invitrogen). For RT-qPCR detection, RNA was reverse-transcribed into cDNA using the HiScript II 1st Strand cDNA Synthesis Kit (Vazyme). Quantitative PCR (qPCR) was performed using diluted cDNA (20× dilution) on a qTOWER3/G real-time PCR machine. *CsaTUB* was used as an internal control for qPCR detection in cucumbers. Primers used are listed in Table S2.

### Luciferase assay

Negative regulation of *CsaWPRa4* by CsamiR396 was investigated using the pGreen II 62-SK/pGreenII 0800-LUC system. The *CsaMIR396A* sequence was amplified and cloned into the pGreen II 62-SK vector between *Bam*H I and *Hin*d III sites using the ClonExpress II One Step Cloning Kit (Vazyme, Nanjing, China) to construct the *35S::CsaMIR396A* vector (effector vector). The 35S promoter and the miR396 binding site of *CsaWPRa4* were amplified and cloned into the pGreenII 0800-LUC vector between *Hin*d III and *Nco* I sites using the ClonExpress II One Step Cloning Kit (Vazyme, Nanjing, China), and then the *35S::CsaWPRa4^miR396 binding site^-LUC* vector (reporter vector) was constructed. Primers used are listed in Table S2.

For the luciferase assay, the control vector (empty vector, pGreen II 62-SK), effector vector (*35S::CsaMIR396A*), and reporter vector (*35S::CsaWPRa4^miR396 binding site^-LUC*) were transformed into Agrobacterium (GV3101). *Nicotiana benthamiana* leaves were infiltrated with transformed Agrobacterium and incubated for 2 days. Thereafter, infected *N. benthamiana* leaves were sprayed with D-Luciferin solution (300 μM) and then imaged by a CCD machine. Additionally, infected *N. benthamiana* leaves were collected to determine the LUC/REN ratio.

### Gain-of-function of AtmiR396a–AtWPRa4 pathway assay

Gain-of-function of the AtmiR396a–AtWPRa4 pathway by synonymous substitutions was investigated using the pGreen II 62-SK/pGreenII 0800-LUC system. For the *35S::AtMIR396A* vector (effector vector), the *AtMIR396A* sequence was amplified and cloned into the pGreen II 62-SK vector between *Bam*H I and *Hin*d III sites using the ClonExpress II One Step Cloning Kit (Vazyme, Nanjing, China). For *35S::AtWPRa4-LUC* vector (reporter vector), the 35S promoter and *AtWPRa4* sequence were amplified and cloned into the pGreenII 0800-LUC vector between *Hin*d III and *Nco* I sites using a ClonExpress II One Step Cloning Kit (Vazyme, Nanjing, China). For the *35S::AtWPRa4m-LUC* vector (reporter vector), the miR396 binding site was substituted by overlapping PCR to obtain the *AtWPRa4m* sequence, and the 35S promoter and *AtWPRa4m* sequence were cloned into the pGreenII 0800-LUC vector between *Hin*d III and *Nco* I sites using the ClonExpress II One Step Cloning Kit (Vazyme, Nanjing, China). Primers used are listed in Table S2.

For the luciferase assay, the effector (*35S::AtMIR396A*) and reporter vectors (*35S::AtWPRa4-LUC* or *35S::AtWPRa4m-LUC*) were transformed into Agrobacterium (GV3101). *Nicotiana benthamiana* leaves were infiltrated with transformed Agrobacterium and incubated for 2 days. Thereafter, infected *N. benthamiana* leaves were sprayed with D-Luciferin solution (300 μM) and then imaged by a CCD machine. Additionally, infected *N. benthamiana* leaves were collected for RT-qPCR analysis of *REN* and *AtWPRa4* expression levels.

### TRSV-based silencing of miR396 in cucumber

TRSV-based gene silencing was performed using the TRSV-CsaGL3 system in cucumbers, as described in a previous study [34], and silencing of miR396 was performed using the short tandem target mimic (STTM) method [35]. Briefly, the STTM fragment for miR396 was prepared and cloned into the *pTRSV2-CsaGL3* vector at the *Sna* BI site using a homologous recombination kit (Vazyme), after which, the *pTRSV2-CsaGL3-STTM396* vector was constructed. Primers used are listed in Table S2.

The vectors were transformed into Agrobacterium (GV3101) and 1-day-old cucumber seedlings were vacuum-agroinfiltrated with GV3101 (*pTRSV1*) + GV3101 (*pTRSV2-CsaGL3*) or GV3101 (*pTRSV1*) + GV3101 (*pTRSV2-CsaGL3-STTM396*). The successfully silenced cucumber seedlings were selected using trichomes as markers and collected for RT-qPCR analysis.

### Subcellular localization prediction and subcellular localization assay

Subcellular localization was predicted using ePlant (https://bar.utoronto.ca/eplant/). For the subcellular localization assay, the coding sequence of *CsaWPRa4* was cloned into the *35S::MCS-eGFP* vector at the *Nco* I site using a homologous recombination kit (Vazyme), and then the *35S::CsaWPRa4-eGFP* vector was constructed. Primers used are listed in Table S2.

The *35S::CsaWPRa4-eGFP* vector was transformed into Agrobacterium (GV3101), and the leaves of *35S::H2B-Red* transgenic *N. benthamiana* were infiltrated with the transformed GV3101 strain and kept for 2 days. Finally, infected *N. benthamiana* leaves were analyzed using a confocal microscope (Olympus).

### Gene overexpression and CRISPR/Cas9-based gene editing in cucumber

To generate *CsaMIR396A-*overexpressing cucumbers, the *CsaMIR396A* sequence was amplified to construct the *35S::CsaMIR396A* vector. To generate CRISPR/Cas9 engineered mutations in the *CsaWPRa4* gene, sgRNA was designed within the first exon of *CsaWPRa4* and cloned into the *pBSbdcas9i* vector. Agrobacterium carrying the *35S::CsaMIR396A* vector or *pBSbdcas9i-CsaWPRa4* vector was used to transform the inbred cucumber line Jinyan using cotyledonary nodes as explants (Biorun BioSciences, Wuhan, China). Transgenic cucumber seedlings were generated, and genomic DNA was extracted for PCR amplification. The *CsaWPRa4* gene was amplified in *Csawpra4* mutants, and various mutant alleles of the *CsaWPRa4* gene were identified by sequencing.

### RNA-seq analysis

Sixteen-day-old WT, *Csawpra4_#1*, and *Csawpra4_#2* seedlings were collected, and total RNAs was extracted using TRIzol reagent (Invitrogen). RNA-seq was performed by METWARE (Wuhan, China). For RNA-seq analysis, DEGs were identified with a value of |log2FC| >1 (fold change of DEGs >2), and the expression level in the WT was used as a reference. Raw transcriptome sequence data were deposited in the NGDC database (https://ngdc.cncb.ac.cn) under the accession number (PRJCA053734).

### Chlorophylls content detection

Fresh cucumber leaves (0.1 g) were homogenized and extracted with 1.4 ml of 95% ethanol solution containing CaCO_3_. The mixture was centrifuged and 1 ml of the supernatant was diluted with 3 ml of 95% ethanol solution (dilution factor = 4). The absorbance of the diluted extract was measured at 665 and 649 nm using a UV–visible spectrophotometer (UV-2550, SHIMADZU, Japan). The chlorophyll a concentration (mg/l) was calculated as 13.36^*^A_665_–5.19^*^A_649_, whereas the chlorophyll b concentration (mg/l) was calculated as 27.43^*^A_649_–8.12^*^A_665_. Chlorophyll content (mg/g) was calculated as C (mg/l) × dilution factor × extraction volume/sample weight.

### Photosynthetic traits detection

Photosynthetic traits were measured using an LI-6800 portable photosynthesis system (LICOR, USA) with a 6800-01F fluorometer leaf chamber (2 cm × 2 cm). Environmental conditions were set as: flow: On; pump speed: Auto; flow rate: 500 μmol/s; H_2_O: On; relative humidity: 65%; CO_2_ injector: On; CO_2_ concentration: 400 μmol/mol; and leaf temperature (Tleaf) maintained at 25°C. Mature cucumber leaves were used to detect the photosynthetic traits.

## Supplementary Material

Web_Material_uhag036

## Data Availability

All experimental data are available in the main text and supplementary data.
