# Supplementary material for: Synonymous substitutions confer the conserved WPRa4 as a novel target of miR396 in cucumber
Source: Hortic Res. 2026 Feb 16;13(5):uhag036. doi: 10.1093/hr/uhag036 (PMC13150849; doi:10.1093/hr/uhag036)
Supplement: Web_Material_uhag036 [file web_material_uhag036.zip › File S1. The information of WPR family members in plants.docx]

**File S1. The information of WPR family members in plants.**

**Fourteen WPR family members in Arabidopsis**

AtPMI15_AT5G38150

AtPMI2_AT1G66840

AtWPRa1_AT1G12150

AtWPRa2_AT3G13190

AtWPRa3_AT4G17210

AtWPRa4_AT5G55860

AtWEB1_AT2G26570

AtWEL1_AT4G33390

AtWEL2_AT1G45545

AtWEL3_AT5G42880

AtWPRb1_AT2G38370

AtWPRb2_AT2G40480

AtWPRb3_AT3G51720

AtWPRb4_AT3G56270

**Nine WPR family members in cucumber**

CsaV3_4G034290

CsaV3_2G027820

CsaWPRa4_CsaV3_3G004170

CsaV3_UNG107770

CsaV3_3G033220

CsaV3_6G002220

CsaV3_1G015450

CsaV3_4G027040

CsaV3_2G033850

**WPRa4 in plants**

AtWPRa4_AT5G55860

GyWPRa4_GlysoPI483463.08G291500

SlWPRa4_Solyc07g065680

OsWPRa4_LOC_Os12g17310

CsaWPRa4_CsaV3_3G004170

CmeWPRa4_MELO3C014113

BhiWPRa4_Bhi11G000369

LsiWPRa4_Lsi05G005040

ClaWPRa4_Cla97C10G188820

CmaWPRa4-1_CmaCh16G009550

CmaWPRa4-2_CmaCh06G006690

TanWPRa4_Tan0005865

SedWPRa4_Sed0007556

LacWPRa4_Lag0017924

MchWPRa4_MC00g0287

SgrWPRa4_tig00153661
