# Supplementary material for: Synonymous substitutions confer the conserved WPRa4 as a novel target of miR396 in cucumber
Source: Hortic Res. 2026 Feb 16;13(5):uhag036. doi: 10.1093/hr/uhag036 (PMC13150849; doi:10.1093/hr/uhag036)
Supplement: Web_Material_uhag036 [file web_material_uhag036.zip › Supplemental Figure 1-7.docx]

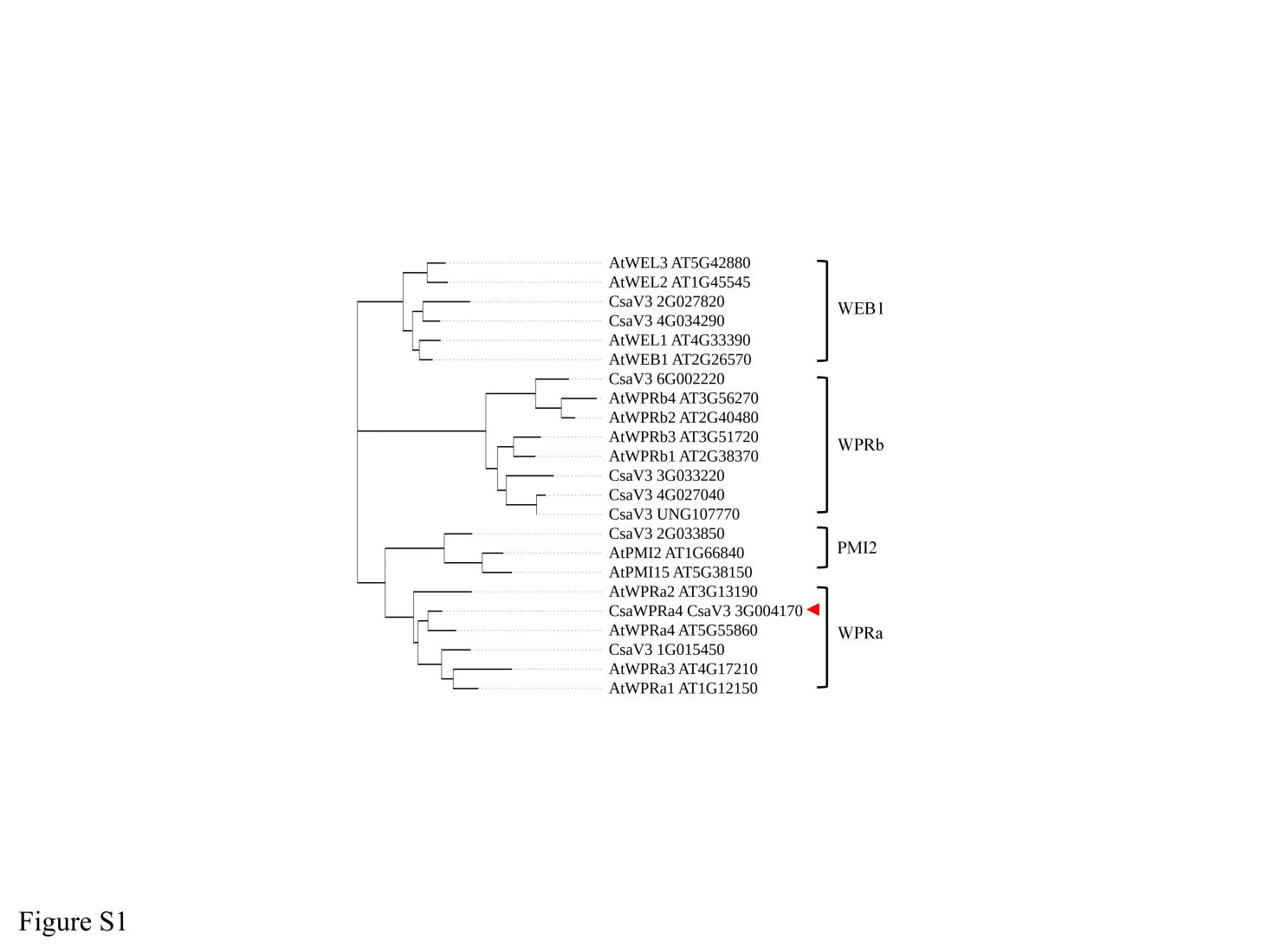


**Figure S1.** Phylogenetic analysis of WPR family members in Arabidopsis and cucumber. Nine WPR family members from cucumber and 14 WPR family members from Arabidopsis are divided into four groups, including the WEB1 group, the PMI2 group, the WPRa group and the WPRb group. CsaWPRa4 is marked with red triangle.


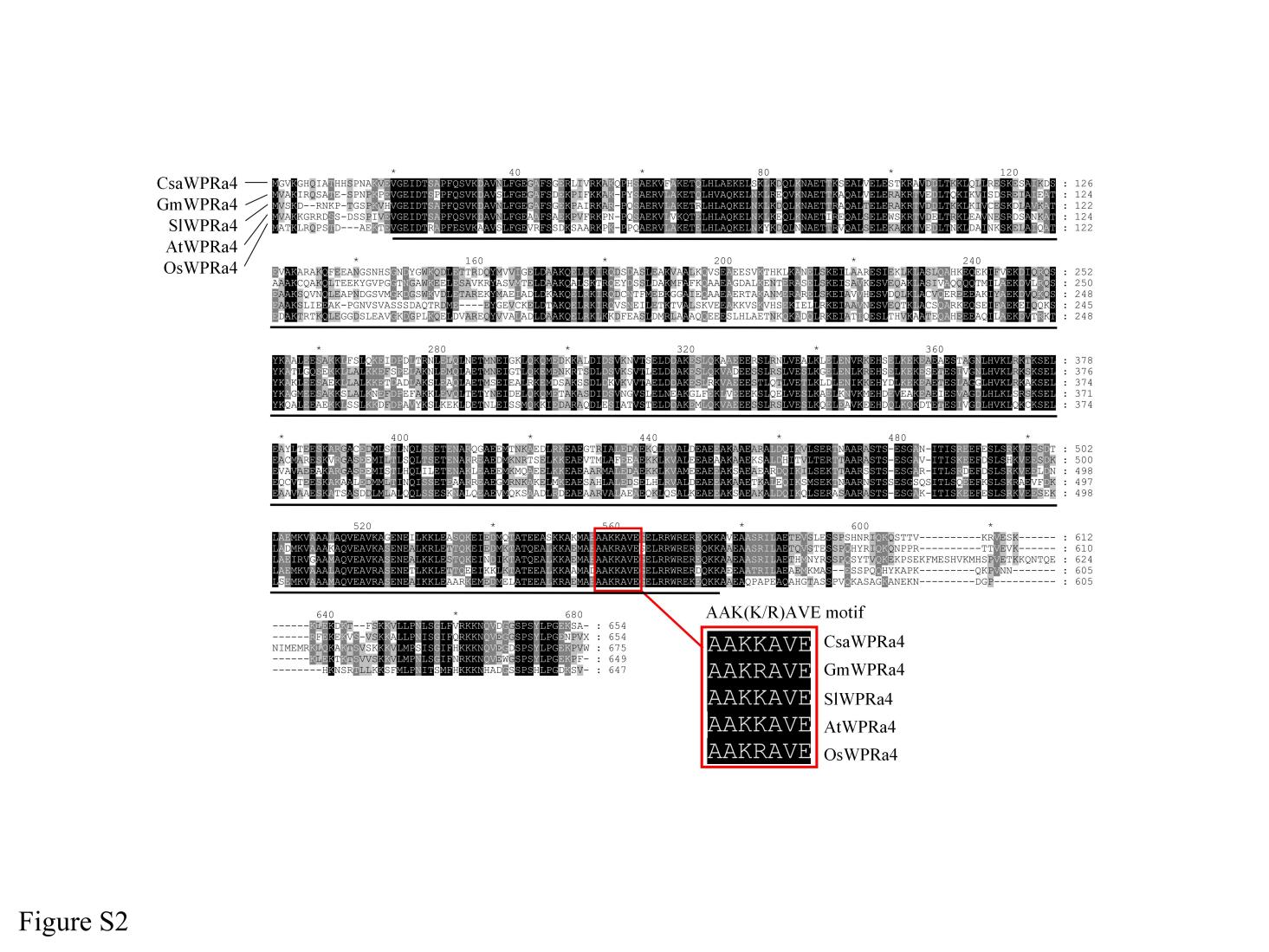


**Figure S2.** Alignment of CsaWPRa4 and its homologs in plants. The WEMBL domain is underlined, while the conserved AAK(K/R)AVE motif is marked with a red box. *Csa*, *Gm*, *Sl*, *At* and *Os* represent *Cucumis sativus*, *Glycine max*, *Solanum lycopersicum*, *Arabidopsis thaliana* and *Oryza sativa*, respectively.

**
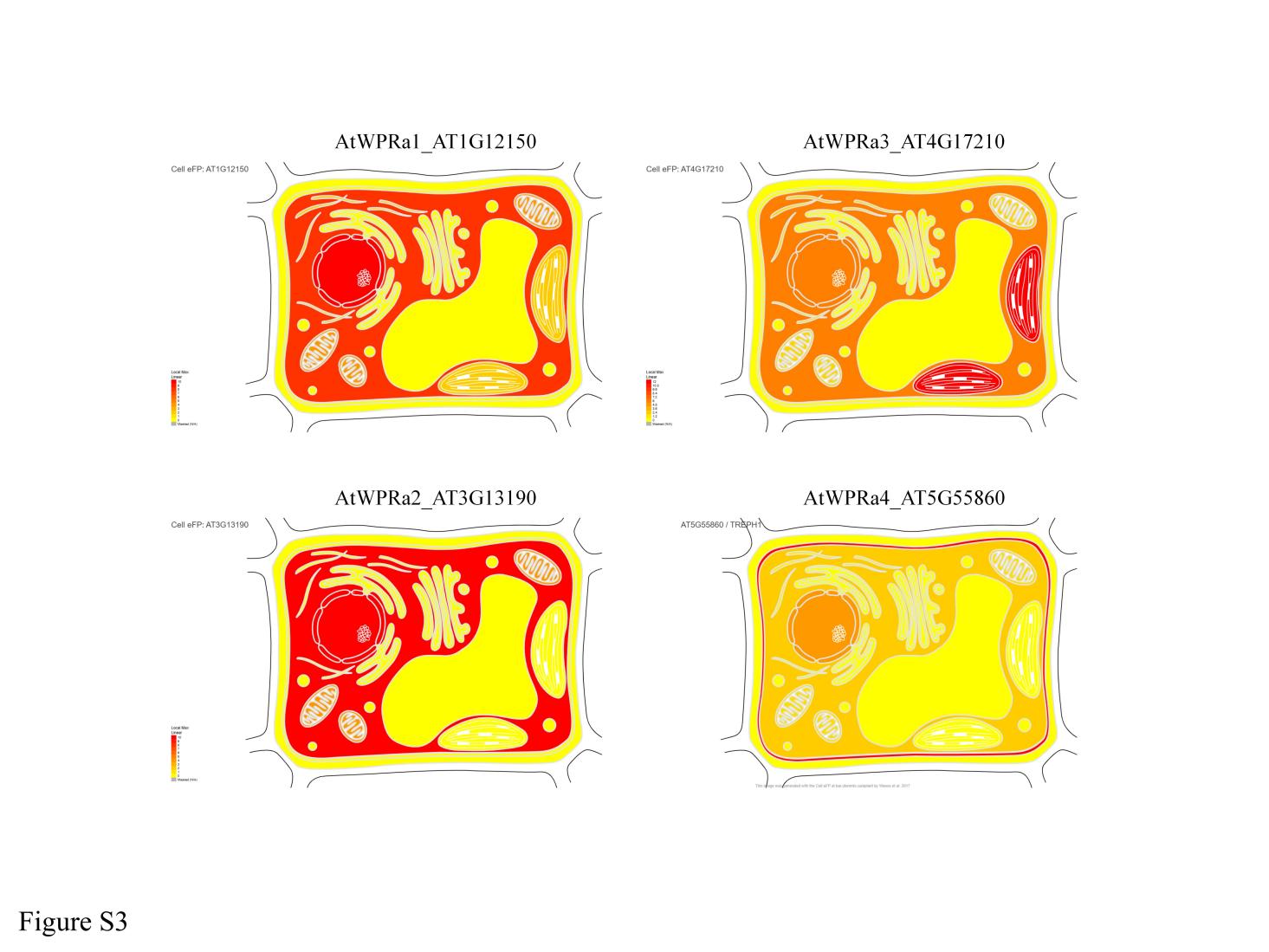
**

**Figure S3.** Predication of the subcellular localization of WPRa family members in Arabidopsis. Subcellular localization prediction performed by ePlant (https://bar.utoronto.ca/eplant/)


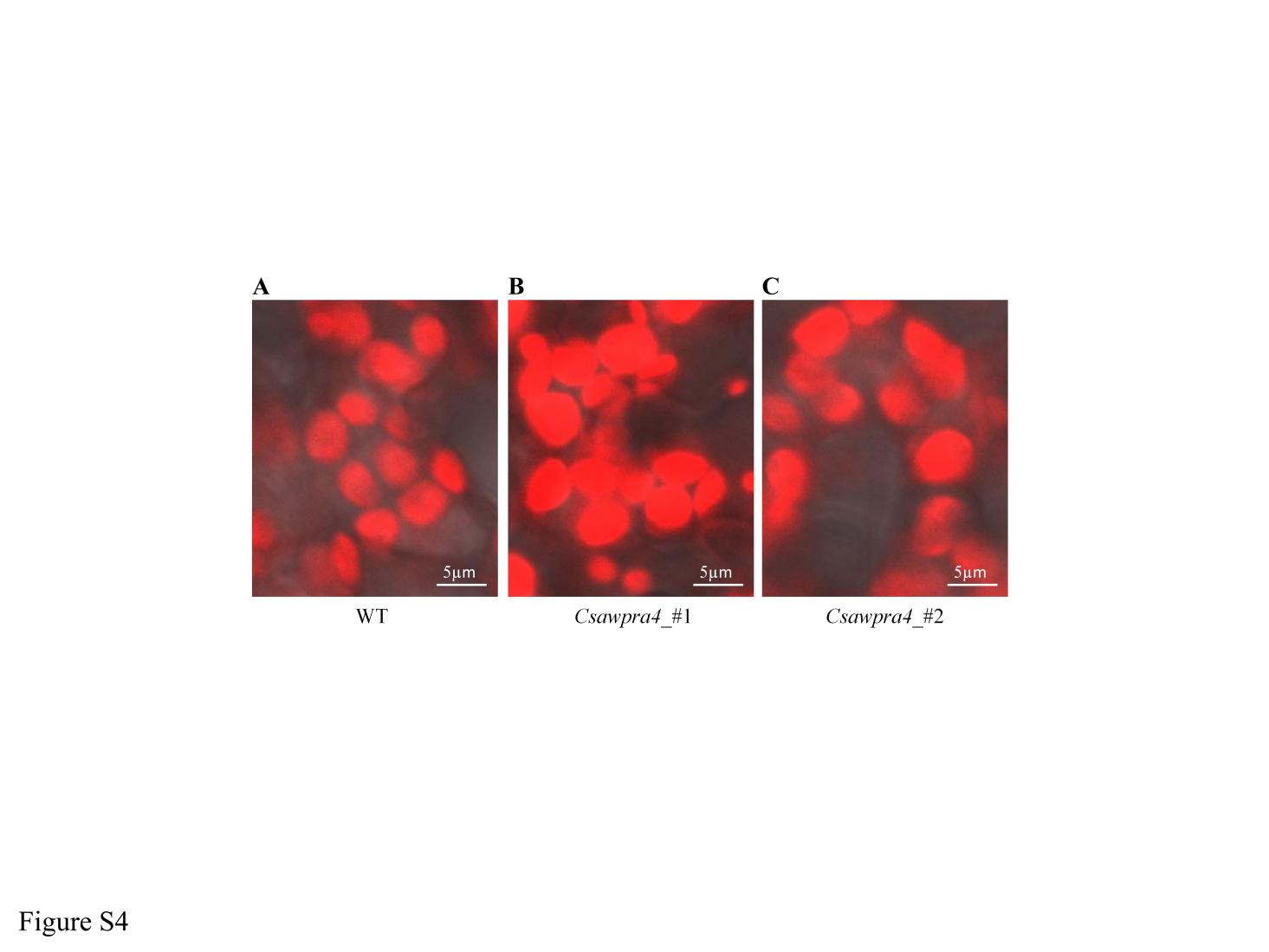


**Figure S4.** The chloroplast of WT and *Csawpra4* mutants. (A) The chloroplast of WT. (B)-(C) The chloroplast of *Csawpra4* mutants.


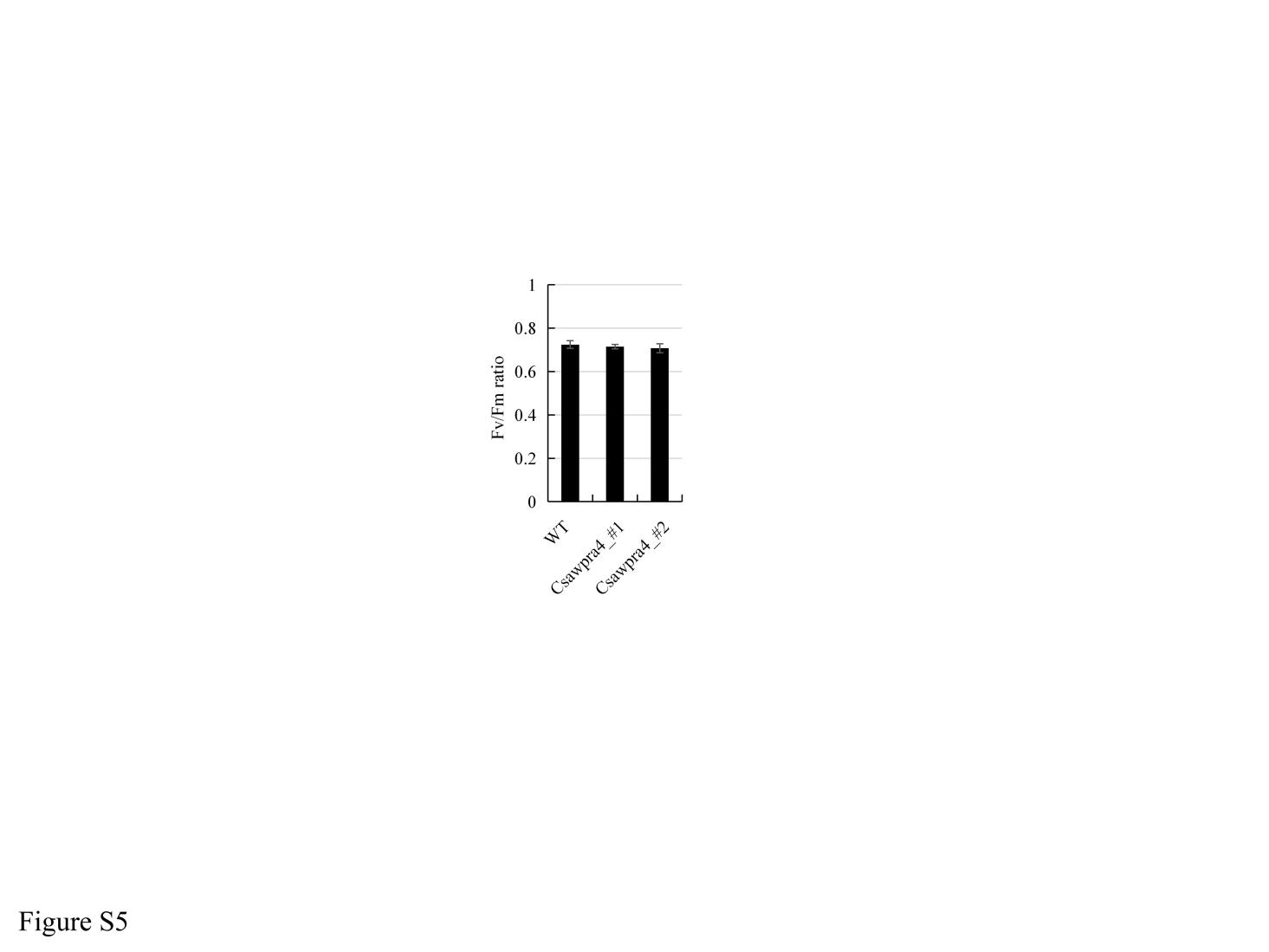


**Figure S5.** Detection of Fv/Fm ratio in WT and *Csawpra4* cucumbers.


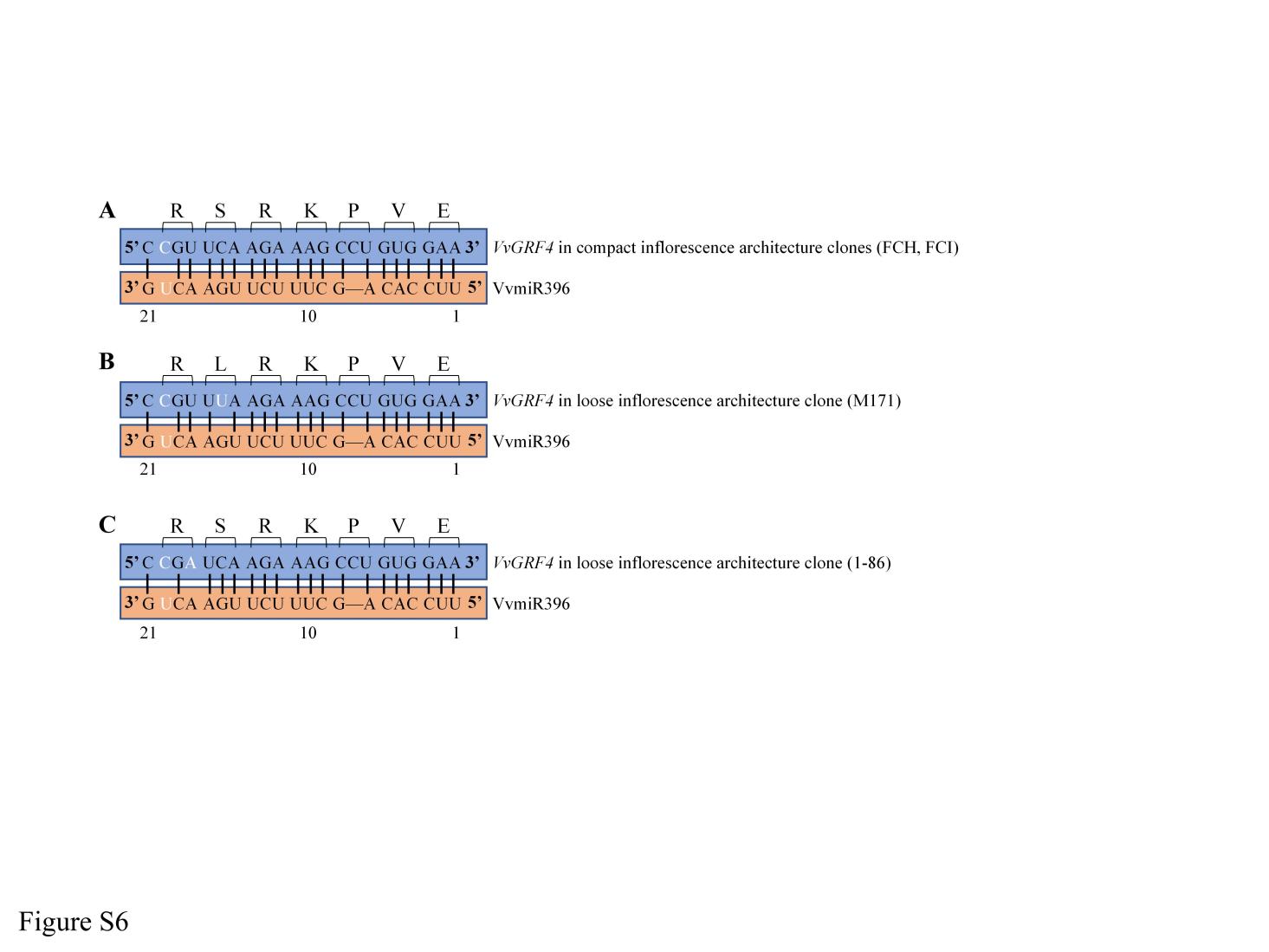


**Figure S6.** Change of miR396 binding site in *VvGRF4* during grapevine domestication. (A) Alignment of miR396 and its binding site in *VvGRF4* in grapevine clones (FCH, FCI) with compact inflorescence architecture. (B) Alignment of miR396 and its binding site in *VvGRF4* in grapevine clone (M171) with loose inflorescence architecture. (C) Alignment of miR396 and its binding site in *VvGRF4* in grapevine clone (1-86) with loose inflorescence architecture. Mismatch nucleotides are marked in white.


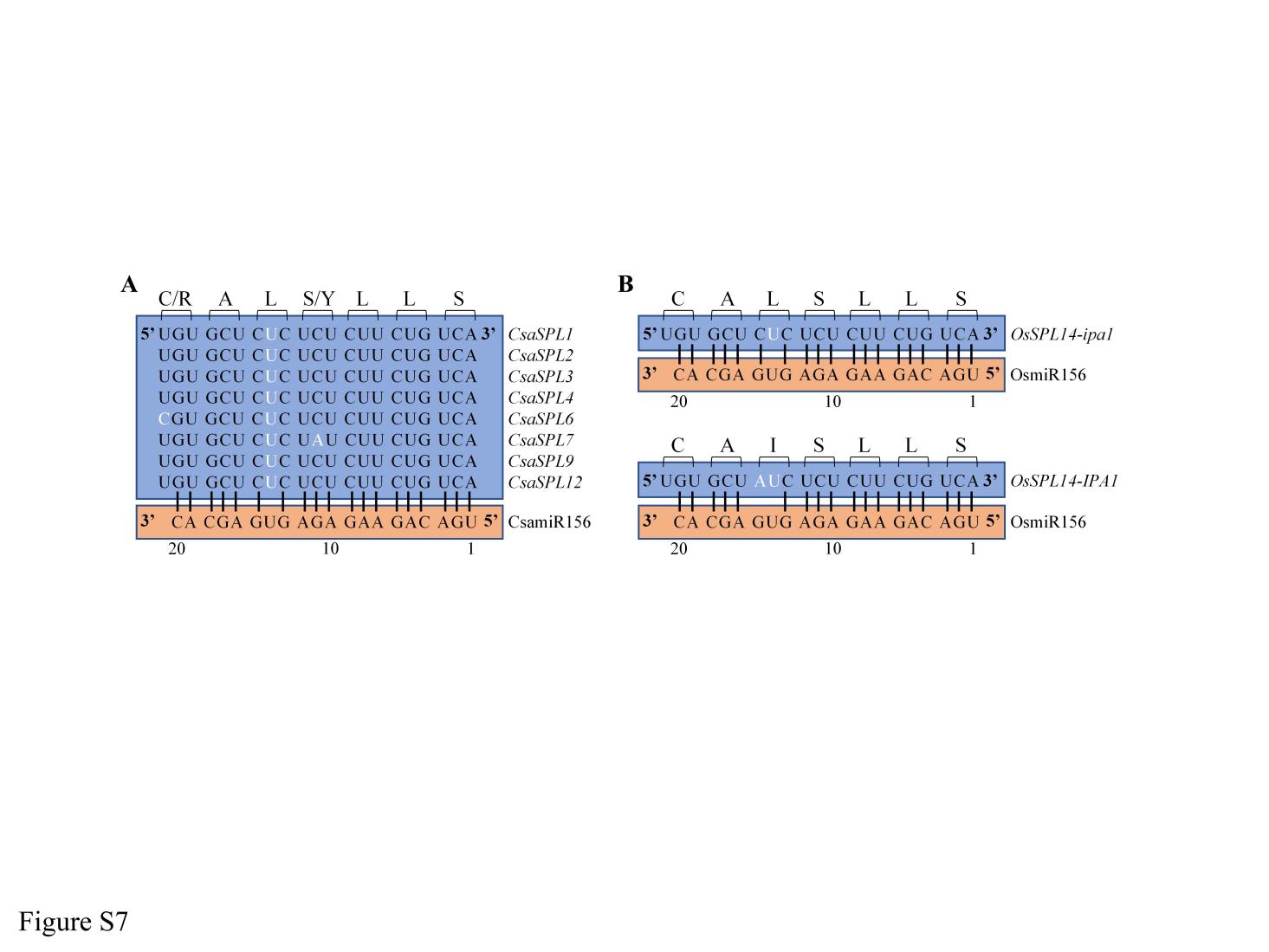


**Figure S7.** Change of miR156 binding site in *SPLs* in cucumber and rice. (A) Alignment of miR156 and its binding site in *CsaSPLs* in cucumber. (B) Alignment of miR156 and its binding site in *OsSPL14* in rice. Mismatch nucleotides are marked in white.
